# 

**Published:** 1895-06

**Authors:** 


Vol. XLIX. JUNE, 1895. No. 6.

### THE DENTAL REGISTER

A MONTHLY JOURNAL.

DEVOTED TO THE INTERESTS OF THE PROFESSION.

EDITED BY

J. TAFT. D.D.S.

ASSOCIATE EDITORS,

WILL. H. WHITSLAR, D.D.S. N. S. HOFF, D.D.S.

**PUBLISHED BY**

SAM'L A. CROCKER & CO.,

SUCCESSORS TO SPENCER & CROCKER,

OHIO DENTAL AND SURGICAL DEPOT,

Nos. 117, 119, 121 WEST FIFTH STREET, CINCINNATI, OHIO.

**TERMS:—$2.00 per Annum, in Advance. Single Copies, 25 cts.**

